# Intimate partner violence as a determinant factor for spontaneous abortion during pregnancy: an unmatched case–control study

**DOI:** 10.3389/fpubh.2023.1114661

**Published:** 2023-06-05

**Authors:** Helen Teweldebrhan Hailu, Wubegzier Mekonnen, Zenawi Hagos Gufue, Selamawit Gebreyohannes Weldegebriel, Berhe Dessalegn

**Affiliations:** ^1^Department of Public Health, College of Medicine and Health Sciences, Adigrat University, Tigray, Ethiopia; ^2^Department of Reproductive Health and Health Service Management, School of Public Health, College of Health Sciences, Addis Ababa University, Addis Ababa, Ethiopia

**Keywords:** intimate partner, violence, pregnancy, spontaneous abortion, Northern Ethiopia

## Abstract

**Background:**

Intimate partner violence affects a significant portion of women worldwide throughout their lifetimes. Ethiopia lacks data that policymakers could utilize to develop context-specific policies for handling intimate partner violence during pregnancy.

**Objectives:**

To identify the determinants of spontaneous abortion among women survivors of intimate partner violence during pregnancy in Adigrat General Hospital, Northern Ethiopia, in 2020.

**Methods:**

A facility based, case–control study design was employed to recruit 371 women (124 cases and 247 controls) attending maternal health services in Adigrat General Hospital, Northern Ethiopia, from March 13 to June 12, 2020. Cases and controls were selected using a consecutive sampling technique. A multivariable binary logistic regression model was carried out to identify potential factors, and a *p*-value of <0.05 was used to declare statistical significance.

**Results:**

The proportion of any form of intimate partner violence during pregnancy among cases and controls was 53.23 and 34.82%, respectively. Any form of intimate partner violence (AOR = 3.66; 95% CI 1.69–7.95), physical intimate partner violence (AOR = 3.06; 95% CI 1.69–7.95), and an interpregnancy interval of <24 months (AOR = 4.46; 95% CI 1.65, 12.07), were the independent determinants of spontaneous abortion among survivors.

**Conclusion:**

Spontaneous abortion was significantly associated with exposure to any form of intimate partner violence, including physical intimate partner violence, and a shorter inter-pregnancy interval.

## Introduction

Intimate partner violence (IPV) is defined as being subjected to physical, emotional, or sexual violence by a current or former partner or spouse (1). Intimate partner violence, is a global public health problem as well as a violation of fundamental human rights. Globally, IPV affects 30% of women in their lifetime, with Africa having the highest-burden accounting for 36.6% (2). According to the Ethiopian Demographic Health Survey (EDHS) 2016 report, the prevalence of IPV among ever-married women in Ethiopia was 34%, with regional differences, 9% in the Ethiopian-Somali region and 38% in the Oromia region (3). Similarly, In Tigray and Afar regions, among women aged 15–49 years who have ever been pregnant, the percentage who have ever experienced violence during pregnancy, was 5.5 and 1.8%, respectively (3).

The physical, sexual, and mental abuse that IPV victims endure during pregnancy has a significant adverse effect on their health as well as the health of their fetuses. Previous reports from the WHO demonstrated a link between the use of IPV during pregnancy and preterm birth, low birth weight, spontaneous abortion, induced abortion, neonatal death, stillbirth, the risk of contracting sexually transmitted diseases (STDs), and poor health service usage (1).

According to a study conducted in Cameroon, women who were exposed to any form of IPV had a 50% higher chance of suffering a spontaneous miscarriage (4). Similarly, studies conducted in Tanzania (5) and Zimbabwe (6) also supported the finding that women who experienced any form of IPV had a higher risk of spontaneous abortion than women who did not experience any form of IPV. However, studies conducted in Ghana (7) and India (8) indicated that women who were exposed to IPV did not have a significant association with early pregnancy loss.

Abortion was responsible for about 7% of maternal deaths and 8.9% of Years of Life Lost (YLL) worldwide in 2017. Similarly, it is anticipated that abortion-related causes account for 8.9% of hospital admissions with almost equal morbidity. Conversely, in Sub-Saharan Africa (SSA), abortion contributed to 9.6% of maternal deaths (9). In Ethiopia, abortion was the fourth-leading cause of maternal mortality from 1990 to 2016 (10). Similarly, 4% of maternal near-miss cases in Addis Ababa, Ethiopia, were due to abortion (11).

Despite the significance of the problem for public health, various studies have produced contradictory findings, and there is a dearth of local epidemiological data. Similarly, there is no adequate evidence that demonstrates whether IPV during pregnancy causes spontaneous abortions in the Tigray region of Northern Ethiopia. By addressing IPV during pregnancy and other risk factors for spontaneous abortion, this research will assist policymakers in developing context-specific prevention strategies. Therefore, the purpose of this study was to identify the determinants of spontaneous abortion among pregnant women who experienced intimate partner violence in Adigrat General Hospital, Northern Ethiopia, in 2020.

## Methods and materials

### Study area and period

The study was conducted at Adigrat General Hospital in the Tigray region of Northern Ethiopia. Adigrat General Hospital is a publicly owned general hospital that also hosts Adigrat University as a teaching hospital. With over 142,765 patient flows annually, it also serves as a referral facility for catchment areas in the Eastern Zone of the Tigray region and some adjacent areas of the Afar region.

The study area was selected based on the Tigray Regional Health Bureau’s 2018 Annual Hospital Report. Adigrat General Hospital had the largest number of inpatient cases of spontaneous abortion, according to the data. Moreover, according to the 2019 Adigrat Hospital report, there were 1,009 abortion cases, 616 of which were spontaneous. The study was conducted between March 13, 2020, and June 12, 2020.

### Study design and participants

A facility-based, case–control study design was used, with the cases (with the condition) consisting of all women who were admitted to the hospital for post-abortion treatment or outpatient care following a spontaneous abortion. Whereas, pregnant women who came to the hospital for their routine third or fourth Antenatal Care (ANC) follow-up visits were considered controls (without the condition). The main reason for restricting those women in their 3rd or 4th ANC visit is to rule out an abortion, as abortion cannot be defined among those women in their 3rd or 4th visits and if we include those pregnant women who were in their 1st or 2nd ANC visits there will be a misclassification bias.

### Sample size and sampling technique

The sample size was determined using double population proportion formula using the Epi info version 7.2.0.1 software for Windows. Taking IPV as a primary exposure variable [26.1% (12)], and with the assumptions of a two-sided significance level (*α* = 5%), power (1-β) = 80, 95% confidence level, a ratio of cases to controls (R) 1:2. When comparing women exposed to IPV to controls, the likelihood of spontaneous abortion was 2.08 (13).

A 15% non-response rate was implemented due to the possibility of unpleasant pregnancy outcomes, the extremely sensitive and private nature of the exposure, and the outcome variable. As a result, the total sample size was expanded to 378, consisting of 126 cases and 252 controls. Until the necessary sample size was reached, all women admitted to post-abortion care and outpatient care services who had spontaneous abortions were contacted using a convenient sampling technique. Similarly, all pregnant women who arrived for their 3rd or 4th ANC follow-up visits were selected until the required sample size was attained.

### Data collection instrument

A pre-tested (in 5% of the population outside of the study area), structured interviewer-administered questionnaire was utilized to collect the variables of interest, and a maternal medical record review was employed to acquire the maternal medical conditions like hypertension status during pregnancy, Human Immunodeficiency Virus (HIV) status, chronic heart disease, diabetes mellitus, and other conditions. The questionnaire was developed after reviewing previous similar studies (14–18). The IPV instrument was adapted from the WHO-2005 multi-country research tool (1) and the EDHS 2016 report (6). The questionnaire was developed in English, then translated into the local language, Tigrigna, and then retranslated back to English by independent professional translators to ensure consistency and understandability of the tool by the women.

The outcome variable of interest was spontaneous abortion and the independent variables were, socio-demographic characteristics, which contain maternal age, maternal education status, marital status, age of at first marriage, residence, religion, occupation, monthly household income, and partner education status. Medical factors include; hypertension during pregnancy, nutritional status of the mother, HIV status (positive/negative), any infectious disease, previously untreated Sexually Transmitted Diseases (STDs), chronic heart disease, and diabetes mellitus.

Maternal reproductive factors include birth interval, history of previous induced abortion, history of previous spontaneous abortion, history of previous cesarean delivery, gestational age, and parity. Risky behavioral factors include substance use during pregnancy, cigarette smoking, khat chewing, alcohol consumption, partner smoke cigarette status, and partner alcohol consumption.

### Data collection process

Three female data collectors with bachelor’s degrees in midwifery who did not work for the hospital, as well as a senior public health professional with a master’s degree, served as a supervisor. The primary investigator trained data collectors two days before the actual data collection period about the study’s objective, the problem’s high level of sensitivity, the confidentiality of responses, and the questionnaires’ contents. Both cases and controls were interviewed using the same study tool. To protect their privacy and encourage their communicative motivations, data were collected in a separate, quiet, private room, and partners were not involved.

### Operational definitions

Spontaneous abortion or miscarriage: defined as the loss of a pregnancy without any intervention before 28 weeks gestational age from the Last Normal Menstrual Period (LNMP) (19).

Emotional IPV during pregnancy is defined as women experiencing any of the following; having been insulted by their husband by using abusive language that made them feel bad; being insulted in front of others; having been scared or intimidated on purpose, or having been threatened by their husband with an object such as a stick, belt, knife, gun, etc. by a current partner or boyfriend during the index pregnancy (1).

Physical IPV during pregnancy is defined as women experiencing any of the following; being slapped or having something thrown at her that could hurt her; being pushed or shoved; being hit with a fist or something else that could hurt; being kicked, dragged, choked, or burned on purpose; and/or being threatened with or having a gun, a knife, or another weapon used on her by a current intimate partner during the index pregnancy (1).

Sexual IPV during pregnancy is defined as women experiencing any of the following; being physically forced to have sexual intercourse when she did not want to, having sexual intercourse because she was afraid of what her partner might do, and/or being forced to do something sexual that she found humiliating or degrading to her by an intimate partner during the index pregnancy (1).

Any IPV during pregnancy is defined as women who experienced at least one of the above offenses being classified as having experienced any IPV during the index pregnancy (1). Risky behavior includes alcohol consumption and khat chewing. Khat (*Catha edulis*) is a plant with psychoactive properties, and the leaves and shoots are chewed (20).

### Data processing and analysis

The completeness and consistency of the obtained data were examined. For data input, Epidata Manager version 4.6.0.2 was utilized, and then the entered data were exported to STATA version 14 for Windows. Descriptive statistics for numeric variables were provided as means (standard deviations) when normally distributed, and as median with interquartile range (IQR) when skewed. Categorical variables were presented using frequency and percentages.

A binary logistic regression model was used to investigate the existence of crude association and to select candidate variables for the final multivariable logistic regression model. Finally, factors with a *p*-value <0.2 (21) were put into a multivariable binary logistic regression model to isolate the effect of IPV on spontaneous abortion, while controlling for other factors. The strength of the association was determined by both the crude odds ratio (COR) and the adjusted odds ratio (AOR) with a 95% confidence interval, and a *p*-value of <0.05 was used to indicate statistical significance.

## Results

### Socio-demographic characteristics

A total of 378 study participants (126 cases and 252 controls) were approached; however, two cases and five controls were not willing to participate. From 371 women (124 cases and 247 controls) completing the interview, the response rate was 98.41% for cases and 98.02% for controls. In the case group, the median age was 26.5 years with an interquartile range of 22 to 32 years. Similarly, the median age of the control group was 26 years, with an interquartile range of 23–30 years. Ninety (72.58%) and 203 (82.19%) of the cases and controls, respectively, were people who lived in cities (Table 1).

**Table 1:** Socio-demographic characteristics of mothers receiving routine maternal health services in Adigrat General Hospital, Northern Ethiopia, 2020 (*n* = 371).

Baseline characteristics		Case (*n* = 124)	Control (*n* = 247)	*p*-value (χ^2^)
		Frequency (%)	Frequency (%)	
Maternal age, median (IQR), years		26.5 (22–32)	26 (23–30)	
Age at first marriage, median (IQR), years		20 (18–23)	20 (19–23)	
Household monthly income, median (IQR), USD		119.58 (89.69, 179.37)	149.48 (89.68, 209.27)	
Maternal age group (years)	<24	38 (30.65)	74 (29.96)	0.12
	25–29	41 (33.06)	106 (42.91)	
	≥30	45 (36.29)	67 (27.13)	
Age at first marriage (Years, *n* = 349)	<18	8 (6.96)	17 (7.26)	0.92
	≥18	107 (93.04)	217 (92.74)	
Place of residence	Urban	90 (72.58)	203 (82.19)	0.03*
	Rural	34 (27.42)	44 (17.81)	
Religion	Orthodox	116 (93.55)	227 (91.9)	0.67§
	Catholic	5 (4.03)	9 (3.64)	
	Others^1^	3 (2.42)	11 (4.45)	
Current marital status	Married	110 (88.71)	232 (93.93)	0.06§
	Single	9 (7.26)	13 (5.26)	
	Others^2^	5 (4.03)	2 (0.81)	
Maternal educational status	No formal education	19 (15.32)	10 (4.05)	<0.001*
	Primary completed	27 (21.77)	33 (13.36)	
	Secondary completed	61 (49.19)	164 (66.4)	
	Higher education	17 (13.71)	40 (16.19)	
Maternal occupational status	Housewife	70 (56.45)	152 (61.54)	0.7
	Farmer	9 (7.26)	12 (4.86)	
	Merchant	15 (12.10)	31 (12.55)	
	Government employee	17 (13.71)	34 (13.77)	
	Daily laborer	7 (5.65)	7 (2.83)	
	Others^3^	6 (4.84)	11 (4.45)	
Partners educational status	No formal education	7 (5.65)	10 (4.05)	0.68
	Primary completed	21 (16.94)	39 (15.79)	
	Secondary completed	67 (54.03)	127 (51.42)	
	Higher education	29 (23.39)	71 (28.74)	
Partner’s occupational status (*n* = 368)	Farmer	21 (17.36)	19 (7.69)	0.04*
	Merchant	27 (22.31)	61 (24.7)	
	Government employee	30 (24.79)	61 (24.7)	
	NGO employee	17 (14.05)	47 (19.03)	
	Carpenter	18 (14.88)	51 (20.65)	
	Others^4^	8 (6.61)	8 (3.24)	
Household monthly income (In United States dollars, *n* = 360)	<$ 44.84	10 (8.55)	20 (8.23)	0.54
	$ 44.84–104.6	32 (27.35)	54 (22.22)	
	≥$ 104.63	75 (64.10)	169 (69.55)	

IQR, interquartile range; NGO, non-governmental organization; USD, United States Dollar.

^1^Includes Protestant and Muslim; ^2^Includes divorced and widowed women; ^3^Includes non-governmental employees and aid-dependent; ^4^Includes daily laborers, jobless, and pensioned. § = *p*-value for Fisher’s exact test, the other *p*-values were for Pearson chi-squared test. *Cases and controls were comparable in their sociodemographic characteristics, except for the maternal place of residence, maternal educational status, and partner’s occupational status (*p* < 0.05).

### Maternal reproductive health characteristics and medical conditions

Regarding the women’s gravidity, 88 (70.97%) cases and 153 (61.94%) controls were both multigravidas. In the case group, 53 (62.35%) women had an inter-pregnancy interval of at least 24 months, whereas in the control group only 122 (84.72%) women had an inter-pregnancy interval of at least 24 months. Regarding the presence of maternal hypertension, five (4.03%) and five (2.02%) of the cases and controls, respectively, had hypertension (Table 2). Regarding the social norm that supports IPV, 23 (18.55%) and 65 (26.32%) of the cases and controls, respectively, stated that there are such norms (Table 2).

**Table 2:** Reproductive health and medical conditions of mothers receiving routine maternal health services in Adigrat General Hospital, Northern Ethiopia, 2020 (*n* = 371).

Baseline variables		Case (*n* = 124)	Control (*n* = 247)	*p*-value (χ^2^)
		Frequency (%)	Frequency (%)	
Average number of total viable pregnancies, median (IQR)		2 (1–4)	2 (1–3)	
Average number of total live births, median (IQR)		2 (1–3)	2 (1–3)	
Inter-pregnancy interval, median (IQR), months		24 (15–36)	36 (24–36)	
Gestational age, median (IQR), weeks		16.04 (± 5.58)^a^	33 (31–35.5)	
MUAC, median (IQR), centimeters		23 (22–24)	23.2 (± 1.66)^b^	
Gravidity	Primigravida	36 (29.03)	94 (38.06)	0.09
	Multigravida	88 (70.97)	153 (61.94)	
Parity (*n* = 241)	≤2	62 (70.45)	107 (69.93)	0.93
	>2	26 (29.55)	46 (30.07)	
Inter-pregnancy interval (Months, *n* = 229)	<24	32 (37.65)	22 (15.28)	<0.001^*^
	≥24	53 (62.35)	122 (84.72)	
MUAC (centimeters)	<21	7 (5.65)	10 (4.05)	0.46
	21–23	69 (55.65)	126 (51.01)	
	>23	48 (38.71)	111 (44.94)	
Previous history of abortion (*n* = 241)	Yes	42 (47.73)	41 (26.8)	0.001^*^
	No	46 (52.27)	112 (73.2)	
Type of abortion (*n* = 83)	Spontaneous abortion	33 (82.5)	38 (88.37)	0.7^§^
	Induced abortion	5 (12.5)	4 (9.3)	
	Both	2 (5)	1 (2.33)	
History of cesarean delivery (*n* = 230)	Yes	12 (14.81)	24 (16.11)	0.8
	No	69 (85.19)	125 (83.89)	
Any unprescribed drug intake during this pregnancy (*n* = 368)	Yes	9 (7.32)	6 (2.45)	0.03^*^
	No	114 (92.68)	239 (97.55)	
Traditional medicine intake during this pregnancy (*n* = 358)	Yes	16 (13.22)	6 (2.53)	<0.001^*^
	No	105 (86.78)	231 (97.47)	
Family history of diabetes mellitus	Yes	5 (4.03)	13 (5.26)	0.6
	No	119 (95.97)	234 (94.74)	
Maternal diabetes mellitus status	Diabetic	0 (0)	1 (0.4)	0.67^§^
	Non-diabetic	124 (100)	246 (99.6)	
Maternal hypertension status	Hypertensive	5 (4.03)	5 (2.02)	0.21^§^
	Non-hypertensive	119 (95.97)	242 (97.98)	
Maternal cardiac disease status	Yes	1 (0.81)	1 (0.4)	0.56^§^
	No	123 (99.19)	246 (99.6)	
STD status during pregnancy	Positive	25 (20.16)	42 (17)	0.46
	Negative	99 (79.84)	205 (83)	
STD treatment during pregnancy (*n* = 63)	Treated	10 (45.45)	18 (43.9)	0.91
	Untreated	12 (54.55)	23 (56.1)	
Maternal serostatus (*n* = 370)	Positive	4 (3.23)	5 (2.03)	0.35^§^
	Negative	120 (96.77)	241 (97.97)	
Social norm that supports IPV	Yes	23 (18.55)	65 (26.32)	0.1
	No	101 (81.45)	182 (73.68)	

IPV, intimate partner violence; IQR, interquartile range; MUAC, mid-upper-arm circumference; STD, sexually transmitted diseases.

^a, b^The gestational age and MUAC values were normally distributed among cases and controls respectively, presented with mean ± standard deviation. § = *p*-value for Fisher’s exact test, the other *p*-values were for Pearson chi-squared test. *Cases and controls were comparable in their reproductive health characteristics and medical conditions, except for the inter-pregnancy interval, previous history of abortion, any unprescribed drug intake, and intake of traditional medicines (*p* < 0.05).

### Risky behavioral factors

From the total number of study participants, 1 (0.81%) cases and 2 (0.81%) controls indicated that they had ever chewed khat. Maternal alcohol intake during the index pregnancy revealed that 79 (83.16%) of cases and 172 (88.21%) of controls had consumed alcohol during their index pregnancies (Table 3).

**Table 3:** Risky behavioral factors of mothers receiving routine maternal health services in Adigrat General Hospital, Northern Ethiopia, 2020 (*n* = 371).

Baseline variables		Case (*n* = 124)	Control (*n* = 247)	*p*-value (χ^2^)
		Frequency (%)	Frequency (%)	
Maternal history of Khat chew	Yes	1 (0.81)	2 (0.81)	0.74^§^
	No	123 (99.19)	245 (99.19)	
Maternal history of cigarette smoked	Yes	1 (0.81)	0 (0)	0.33^§^
	No	123 (99.19)	247 (100)	
Current status of tobacco smoke	Smoker	0 (0)	1 (0.4)	0.67^§^
	Non-smoker	124 (100)	246 (99.6)	
Ever drink alcohol	Yes	98 (79.03)	194 (78.54)	0.91
	No	26 (20.97)	53 (21.46)	
Alcohol intake during the current pregnancy (*n* = 290)	Yes	79 (83.16)	172 (88.21)	0.24
	No	16 (16.84)	23 (11.79)	
Frequency of drinking alcohol (*n* = 255)	Few times per week	20 (24.1)	22 (12.79)	0.02*
	On special occasions	63 (75.9)	150 (87.21)	
Partner chew khat	Yes	4 (3.23)	6 (2.43)	0.28^§^
	No	107 (86.29)	226 (91.5)	
	I do not know	13 (10.48)	15 (6.07)	
Partner smoke cigarettes	Yes	9 (7.26)	8 (3.24)	0.18
	No	108 (87.1)	228 (92.31)	
	I do not know	7 (5.65)	11 (4.45)	
Partner drink alcohol	Yes	97 (78.23)	210 (85.02)	0.17
	No	21 (16.94)	32 (12.96)	
	I do not know	6 (4.84)	5 (2.02)	
Frequency of partner drinking alcohol (*n* = 306)	Daily	13 (13.4)	19 (9.09)	0.42
	Few times a week	68 (70.1)	147 (70.33)	
	On special occasions	16 (16.49)	43 (20.57)	

§ = *p*-value for Fisher’s exact test, the other *p*-values were for Pearson chi-squared test. *Cases and controls were comparable in their risky behavioral factors, except for the Frequency of drinking alcohol (*p* < 0.05).

### The magnitude of intimate partner violence during pregnancy

**Figure 1:**
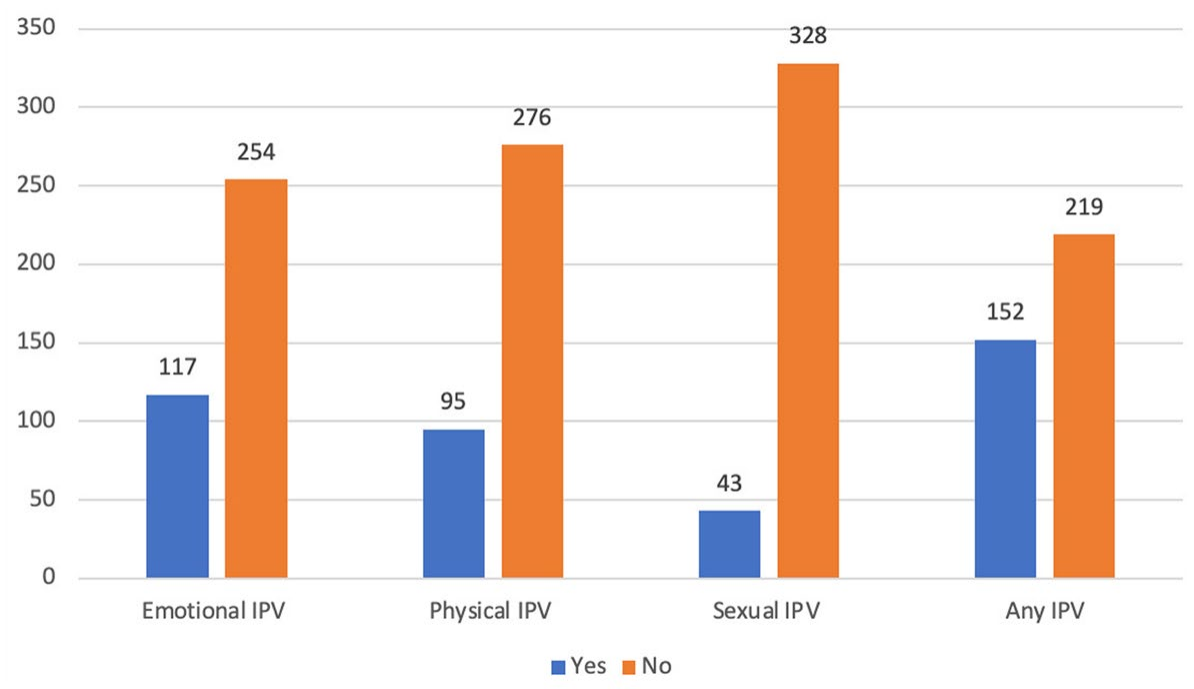
Intimate partner violence during pregnancy among mothers receiving routine maternal health services in Adigrat General Hospital, Northern Ethiopia, 2020 (*n* = 371). IPV, intimate partner violence.

The total percentage of pregnant women who were exposed to any form of IPV was 152 (40.97%), with 66 (53.23%) cases and 86 (34.82%) controls. In both the cases and the controls, the percentage of women who experienced physical IPV during the index pregnancy was 48 (38.71%) and 47 (19.03%) among cases and controls, respectively (Figure 1).

### Determinants of spontaneous abortion

Bivariate analysis was carried out to identify those candidate variables for the final model, having a *p*-value <0.2 (Table 4). Two models were used for identifying the determinants of spontaneous abortion, where model one contains any form of IPV and all other variables, whereas model two contains specific types of IPV (we did not include any form of IPV here) and all other variables. Accordingly, after adjusting for all other factors, women who were exposed to any form of IPV during pregnancy were almost four times more likely to have a spontaneous abortion than those who were not exposed to any form of IPV (AOR = 3.66; 95% CI 1.69–7.95).

**Table 4:** Bivariate analysis for determining the candidate variables for the final model among mothers receiving routine maternal health services in Adigrat General Hospital, Northern Ethiopia, 2020 (*n* = 371).

Variables		Cases (*n* = 124)	Controls (*n* = 247)	COR	*p*-value
		Frequency (%)	Frequency (%)		
Emotional IPV	No	70 (56.45)	184 (74.49)	Ref	
	Yes	54 (43.55)	63 (25.51)	2.25	<0.0001
Physical IPV	No	76 (61.29)	200 (80.97)	Ref	
	Yes	48 (38.71)	47 (19.03)	2.69	<0.0001
Sexual IPV	No	103 (83.06)	225 (91.09)	Ref	
	Yes	21 (16.94)	22 (8.91)	2.09	0.03
Any IPV	No	58 (46.77)	161 (65.18)	Ref	
	Yes	66 (53.23)	86 (34.82)	2.13	0.01
Maternal age (years)	≥30	45 (36.29)	67 (27.13)		
	<24	38 (30.65)	74 (29.96)	1.31	0.33
	25–29	41 (33.06)	106 (42.91)	1.74	0.04
Age at first marriage (years)	≥18	107 (93.04)	217 (92.74)		
	<18	8 (6.96)	17 (7.26)	1.05	0.92
Residence	Rural	34 (27.42)	44 (17.81)		
	Urban	90 (72.58)	203 (82.19)	1.74	0.03
Marital status	Currently unmarried	14 (11.29)	15 (6.07)		
	Currently married	110 (88.71)	232 (93.93)	1.97	0.08
Maternal educational status	Higher	17 (13.71)	40 (16.19)		
	No formal education	19 (15.32)	10 (4.05)	0.22	0.01
	Primary completed	27 (21.77)	33 (13.36)	0.52	0.1
	Secondary completed	61 (49.19)	164 (66.4)	1.14	0.68
Partner educational status	Higher	29 (23.39)	71 (28.74)		
	No formal education	7 (5.65)	10 (4.05)	0.58	0.32
	Primary completed	21 (16.94)	39 (15.79)	0.76	0.43
	Secondary completed	67 (54.03)	127 (51.42)	0.77	0.34
Maternal occupational status	Non-housewife	54 (43.55)	95 (38.46)		
	Housewife	70 (56.45)	152 (61.54)	1.23	0.35
Partner occupational status	Government employee	30 (24.79)	61 (24.7)		
	Non-government employee	91 (75.21)	186 (75.3)	1.01	0.98
Household monthly income (in USD)	≥$ 104.63	75 (64.10)	169 (69.55)		
	<$ 44.84	10 (8.55)	20 (8.23)	0.89	0.77
	$ 44.84–104.6	32 (27.35)	54 (22.22)	0.75	0.27
Parity	≤2	62 (70.45)	107 (69.93)		
	>2	26 (29.55)	46 (30.07)	1.03	0.93
Inter-pregnancy interval (In months)	≥24	53 (62.35)	122 (84.72)		
	<24	32 (37.65)	22 (15.28)	3.35	<0.0001
History of abortion	Yes	42 (47.73)	41 (26.8)		
	No	46 (52.27)	112 (73.2)	2.49	0.01
History of cesarean section	No	69 (85.19)	125 (83.89)		
	Yes	12 (14.81)	24 (16.11)	1.1	0.8
Traditional medicine intake	No	105 (86.78)	231 (97.47)		
	Yes	16 (13.22)	6 (2.53)	5.87	<0.0001
Ever drink alcohol	No	26 (20.97)	53 (21.46)		
	Yes	98 (79.03)	194 (78.54)	1.03	0.91
Alcohol intake during the current pregnancy	No	16 (16.84)	23 (11.79)		
	Yes	79 (83.16)	172 (88.21)	1.51	0.24
Frequency of drinking alcohol	Few times per week	20 (24.1)	22 (12.79)		
	On special occasions	63 (75.9)	150 (87.21)	2.16	0.03
MUAC (centimeters)	<21	7 (5.65)	10 (4.05)		
	21–23	69 (55.65)	126 (51.01)	1.28	0.63
	>23	48 (38.71)	111 (44.94)	1.62	0.36
STD status during pregnancy	Positive	25 (20.16)	42 (17)		
	Negative	99 (79.84)	205 (83)	1.23	0.46
Maternal serostatus	Positive	4 (3.23)	5 (2.03)		
	Negative	120 (96.77)	241 (97.97)	1.61	0.49
Social norm that supports IPV	No	101 (81.45)	182 (73.68)		
	Yes	23 (18.55)	65 (26.32)	1.57	0.1

COR, crude odds ratio; MUAC, mid-upper-arm-circumference; IPV, intimate partner violence; Ref, reference group; STD, sexually transmitted diseases; USD, United States Dollar.

Similarly, pregnant women who were physically exposed to IPV had a threefold higher chance of spontaneous abortion than pregnant women who were not physically exposed to IPV (AOR = 3.06; 95% CI 1.49–6.63). Moreover, in models one and two, women who had an interpregnancy interval (IPI) of <24 months experienced a four-fold increase in the risk of spontaneous abortion compared to those who had an IPI of >24 months (AOR = 4.46; 95% CI 1.65, 12.07), and (AOR = 4.21; 95% CI 1.57, 11.29), respectively (Table 5).

**Table 5:** Determinants of spontaneous abortion among mothers receiving routine maternal health services in Adigrat General Hospital, Northern Ethiopia, 2020 (*n* = 371).

Variables		Cases (*n* = 124)	Controls (*n* = 247)	Model 1	Model 2
		Frequency (%)	Frequency (%)	AOR (95% CI)	AOR (95% CI)
Emotional IPV	No	70 (56.45)	184 (74.49)		Ref
	Yes	54 (43.55)	63 (25.51)		1.49 (0.6–3.65)
Physical IPV	No	76 (61.29)	200 (80.97)		Ref
	Yes	48 (38.71)	47 (19.03)		3.06 (1.49–6.63)**
Sexual IPV	No	103 (83.06)	225 (91.09)		Ref
	Yes	21 (16.94)	22 (8.91)		2.03 (0.58–7.11)
Any IPV	No	58 (46.77)	161 (65.18)	Ref	
	Yes	66 (53.23)	86 (34.82)	3.66 (1.69–7.95)**	
Maternal age (years)	≥30	45 (36.29)	67 (27.13)	Ref	
	<24	38 (30.65)	74 (29.96)	1.07 (0.35–3.3)	0.89 (0.29–2.75)
	25–29	41 (33.06)	106 (42.91)	1.72 (0.75–3.91)	1.54 (0.66–3.6)
Residence	Rural	34 (27.42)	44 (17.81)	Ref	
	Urban	90 (72.58)	203 (82.19)	1.22 (0.48–3.09)	1.23 (0.48–3.12)
Marital status	Currently unmarried	14 (11.29)	15 (6.07)	Ref	
	Currently married	110 (88.71)	232 (93.93)	2.19 (0.25–19.36)	2.41 (0.27–21.84)
Maternal educational status	Higher	17 (13.71)	40 (16.19)	Ref	
	No formal education	19 (15.32)	10 (4.05)	0.57 (0.13–2.61)	0.62 (0.14–2.77)
	Primary completed	27 (21.77)	33 (13.36)	0.89 (0.26–3.06)	0.95 (0.27–3.27)
	Secondary completed	61 (49.19)	164 (66.4)	1.61 (0.53–4.94)	1.66 (0.54–5.06)
Inter-pregnancy interval (In months)	≥24	53 (62.35)	122 (84.72)	Ref	
	<24	32 (37.65)	22 (15.28)	4.46 (1.65–12.07)**	4.21 (1.57–11.29)**
History of abortion	Yes	42 (47.73)	41 (26.8)	Ref	
	No	46 (52.27)	112 (73.2)	1.05 (0.43–2.53)	0.98 (0.4–2.4)
Traditional medicine intake	No	105 (86.78)	231 (97.47)	Ref	
	Yes	16 (13.22)	6 (2.53)	2.28 (0.52–10.03)	2.67 (0.58–12.21)
Frequency of drinking alcohol	Few times per week	20 (24.1)	22 (12.79)	Ref	
	On special occasions	63 (75.9)	150 (87.21)	1.35 (0.53–3.41)	1.62 (0.65–4.04)
Social norm that supports IPV	No	101 (81.45)	182 (73.68)	Ref	
	Yes	23 (18.55)	65 (26.32)	2.35 (0.9–6.15)	2.28 (0.87, 6)

AOR, adjusted odds ratio; CI, confidence interval; COR, crude odds ratio; IPV, intimate partner violence; Ref, reference group.

**Statistical significance at *p* < 0.01.

## Discussion

In this study, we tried to determine the determinants of spontaneous abortion among IPV survivors in Northern Ethiopia. This study found that women who experienced any kind of IPV during their pregnancies had almost a fourfold increased risk of spontaneous abortion compared to women who did not experience any kind of IPV. Similarly, a study carried out in Ethiopia revealed that women exposed to any kind of IPV had a 54% higher chance of having an abortion than those who had not been exposed to any form of IPV (22).

Our study revealed that women who experienced physical IPV during pregnancy were three times more likely to experience a spontaneous abortion than women who did not. This result was consistent with the Nigerian study, which found that women who were physically exposed to IPV were twice as likely to spontaneously abort their children as women who were not (13). Similar to this, Ghanian women who were exposed to physical IPV had a four times greater chance of spontaneous abortion than those who were not exposed to physical IPV (23).

This difference can be explained by the difference in the sociocultural discrepancy across the study settings, and these studies measured the exposure to physical IPV using 1 year or lifetime exposure to physical IPV, which leads to an under- or overestimation of the impact. Studies undertaken in the Democratic Republic of the Congo (24) and India (11), in contrast to our findings, revealed that there was no conclusive link between physical IPV and spontaneous abortion.

This may occur as a direct result of physical injury to the abdomen, which will harm the pregnancy and lead to blood loss and fetus loss. The second method is indirect physical and emotional IPV can impact pregnancy because stress and emotional trauma raise cortisol levels, which cause blood vessels to constrict and reduce blood flow to the uterus (5).

Compared to women who had an IPI of at least 24 months, women with an IPI of less than 24 months were four times more likely to spontaneously terminate their unborn child. Spontaneous abortion was nearly two times more likely to occur in Bangladeshi women with IPIs of less than 14 months (25). Long gaps between pregnancies may affect spontaneous abortion because the uterus needs more time to heal after childbirth or miscarriage. This could serve as a cue to encourage the use of family planning. Health professionals should recommend women have at least 24 months of inter-pregnancy intervals.

Sexual IPV during pregnancy and spontaneous abortion had no statistically significant relationship. Our findings were consistent with the study conducted in the Congo (16), which showed that women who had experienced sexual IPV did not significantly increase their risk of spontaneous abortion. Contradicting this, meanwhile, was a study from Cameroon (7) that found women exposed to a sexual type of IPV had 60% higher risks of spontaneous abortion than those who were not exposed to sexual IPV.

The reasons for this could include variations in study design, methods used to evaluate exposure times, underreporting of sexual IPV during pregnancy, and sociocultural differences between study locations. Even though there was no statistical significance, sexual IPV during pregnancy could disrupt social relationships and result in the isolation of women from family and friends. It can also result in emotional distress for women in their daily routine activities, so psychiatrists, and psychologists should support survivors of IPV for better recovery from the trauma.

### Strengths and limitations of the study

The study’s strength was that it was the first local study to date to use the WHO’s standard questionnaires to evaluate the outcome variable and seek to demonstrate the association between intimate partner violence and spontaneous abortion. Due to the sensitivity of the subject, there are certain study limitations, and responses to intimate partner abuse during pregnancy may still be underreported.

Another limitation was the possibility that an induced abortion could have been mistaken for a spontaneous one, which could have led to an inaccurate assessment of the outcome. Since the study is facility-based using convenient sampling, generalizing the findings is difficult. We did not specifically address those types of social norms that support IPV; listing and determining those specific social norms that support IPV would have been important. Therefore, it is important to consider such potential limitations when interpreting the findings of this study.

## Conclusion

In conclusion, our study demonstrated a strong relationship between spontaneous abortion and exposure to any type of intimate partner violence, including physical intimate partner violence during pregnancy, as well as a shorter inter-pregnancy gap. Intimate partner violence legal framework implementation should be strengthened, as per our recommendation to policymakers.

## Author’s note

The abstract of this paper was presented to Addis Ababa University as a thesis talk with interim findings. The data is available in the institutional repository of Addis Ababa University. Available at: http://213.55.95.56/bitstream/handle/123456789/25012/Helen%20Teweldebrhan.pdf?sequence=1&isAllowed=y.

## Data availability statement

The datasets presented in this study can be found in online repositories. The names of the repository/repositories and accession number(s) can be found at: http://213.55.95.56/bitstream/handle/123456789/25012/Helen%20Teweldebrhan.pdf?sequence=1&isAllowed=y.

## Ethics statement

The studies involving human participants were reviewed and approved by Ethical approval was obtained from the Research and Ethics Committee (REC) of the School of Public Health, College of Health Sciences, Addis Ababa University (Ref: SPH/033/2020). Written informed consent was obtained from each participant, and the data collected from the women and medical records were handled with strong confidentiality; neither the case records nor the collected data were used for any other purpose. All the collected patient information was stored anonymously, and the study was conducted following the 1964 Declaration of Helsinki. The patients/participants provided their written informed consent to participate in this study.

## Author contributions

HH and SW: project inception, management, and clinical input. WM and SW: project inception and questionnaire design. ZG, HH, and BD conducted the statistical analysis and interpreted the findings. All authors have read and approved the final manuscript.

## Conflict of interest

The authors declare that the research was conducted in the absence of any commercial or financial relationships that could be construed as a potential conflict of interest.

## Publisher’s note

All claims expressed in this article are solely those of the authors and do not necessarily represent those of their affiliated organizations, or those of the publisher, the editors and the reviewers. Any product that may be evaluated in this article, or claim that may be made by its manufacturer, is not guaranteed or endorsed by the publisher.
